# Diversity and Evolution of HIV and HCV

**DOI:** 10.3390/v13040642

**Published:** 2021-04-09

**Authors:** Miguel Angel Martinez

**Affiliations:** IrsiCaixa, Hospital Universitari Germans Trias i Pujol, 08916 Badalona, Spain; MMartinez@irsicaixa.es

In this Special Issue focused on human immunodeficiency virus (HIV) and hepatitis C virus (HCV) diversity and evolution, we can find good examples of how the genetic variability of these two viruses is impacting their spread, pathogenesis, and therapeutics. Yeo et al. review the high mutation rate of HIV, which is estimated in a large range of 10^−5^ to 10^−3^ errors/base-pair/cycle. Interestingly and similar to other RNA viruses, the HCV mutation rate is very similar to that found with HIV (Tisthammer et al.). Martinez and Franco also review how HCV mutation rates and diversity have impacted the virus diversity and virus therapeutic control. What we know about HIV and HCV mutation rates can serve as a good model for the comprehensive study of emerging viral diseases, such as the new human coronaviruses or the Ebola virus. Gelbart et al. study on the site-specific evolutionary rate shifts in HIV and simian immunodeficiency virus (SIV). This paper exemplifies the constraints operating in proteins during cross-species transmission, which is also crucial to understand the emergence of new human viruses. Di Giallonardo et al. give another example of the way viruses diversify. These authors show how the changes over time in HIV diversity shape local virus epidemics. In addition to the study of in vivo HIV diversification, Kapaata et al. show that the diversity across the HIV structural Gag and enzymatic Pol protein regions are frequently associated with a higher virus replication capacity in tissue culture. These findings may have implications in understanding HIV infectivity and pathogenesis. Two additional studies by Colomba et al. and Liu et al. describe new data about the impact of HCV and HIV diversification on therapy resistance and failure. Finally, Gracia-Crespo et al. develop a model to explain why HCV population sequence space can be in disequilibrium even in the absence of external selective constraints or changes in population size. This persistent disequilibrium may facilitate alternative mutational pathways for HCV. Importantly, we may apply this model to other high variable viruses. In short, this Viruses Special Issue provides a comprehensive analysis of HIV and HCV variability, particularly focusing on global virus diversification and its impact on antiviral therapy. Our experience with previous emerging human viral pandemics, such as HIV and HCV, should help in understanding and fighting new virus threats.

